# Navigating extreme class imbalance in suicide risk prediction

**DOI:** 10.3389/fpsyt.2025.1679618

**Published:** 2026-01-12

**Authors:** Christopher Kitchen, Anas Belouali, Paul S. Nestadt, Holly C. Wilcox, Hadi Kharrazi

**Affiliations:** 1 Center for Population Health Information Technology (IT), Johns Hopkins School of Public Health (JHSPH), Baltimore, MD, United States; 2 Section of Biomedical Informatics and Data Science, Johns Hopkins School of Medicine (JHSOM), Baltimore, MD, United States; 3 Psychiatry and Behavioral Sciences, Johns Hopkins School of Medicine (JHSOM), Baltimore, MD, United States; 4 Center for Suicide Research, Johns Hopkins School of Public Health (JHSPH), Baltimore, MD, United States

**Keywords:** suicide risk estimation, classification metrics, class imbalance, machine learning, clinical decision support

## Abstract

**Background:**

The implementation of suicide risk models is challenging because the conditions in which they are developed often do not reflect those in which they are being used. The setting of an arbitrary classification threshold limits the interpretability of predictions, and their associated performance statistics. This work endeavors to explore different class imbalance ratios, across training sample compositions, time horizons and patient characteristics to understand how degree of imbalance affects the associated performance of regression-based predictive models of suicide.

**Patients and setting:**

The study population included 1,649,577 patients who were selected from the Maryland Suicide Data Warehouse (MSDW) between 2016 and 2020. The MSDW contains clinical and demographic features derived from claims (Maryland Health Care Commission, MHCC)and hospital discharge records Health Services Cost Review Commission (HSCRC), for decedents and living patients within the state of Maryland. Suicide death was our primary outcome of interest in a cross validated framework stratified by sources of data in the MSDW.

**Results:**

Cross validated AUROC was not found to vary consistently with respect to training sample imbalance nor time horizon, but both were found to have a direct association with AUPRC. Indeed, AUPRC increased with greater sample imbalance for training or outcome horizon (AUPRC 0.246; 0.246; 0.593 for all decedents, HSCRC, and MHCC respectively). Stratified samples revealed no significant cross validated performance than the overall sample for AUROC (0.832; 0.913; 0.927, for decedents, HSCRC and MHCC). However, AUPRC was significantly greater when limiting our HSCRC and MHCC samples to patients seen in the emergency room (AUPRC 0.417; 0.782) or in the inpatient settings (0.371; 0.773), or patients who had ICD-10-CM coded social needs (0.479, HSCRC only). Performance was significantly worse when restricting samples to patients aged less than 18 years (AUPRC 0.036; 0.208, HSCRC and MHCC respectively).

**Conclusion:**

A low precision for estimated suicide risk can be understood as a consequence of some tradeoffs during model development, particularly training models with matched cases, balanced classes or within short time horizons. This work demonstrates the improved AUPRC performance of regression models in a cross validated framework when these conditions are made more realistic, in the context of class imbalance or less restrictive in that of time horizon. Additionally, we illustrate using the same data that training models in certain clinical cohorts (e.g., defined by age, care utilization and social need) can lead to robustly different estimates for precision and recall, but not AUROC.

## Introduction

Clinical researchers often struggle with implementing suicide risk and prevention models in health care settings as these prediction models often lack adequate practical precision (1–3). Clinicians also have a difficult time with such predictive tools for decision support, both because of alert fatigue and a lack of clarity in how models score risk (4). Although some suicide prediction models have yielded clinically reasonable performances, such models are designed for narrow use cases among specific subpopulations (e.g., patients with chronic psychiatric conditions, substance use disorders, or military veterans) (5–8). Indeed, despite a few studies that suggest existing tools are sufficiently actionable, and multiple systematic reviews trying to make sense of very diverse methods of development, a general risk score to accommodate all patients and settings with sufficient precision and sensitivity remains elusive (9–13).

The assessment of suicide risk, like most clinical support tasks, constitutes an imbalanced classification problem. A severe case of imbalance exists when generalizable, health system-wide suicide risk prediction is attempted, leaving us with fewer options for fairly assessing performance in observational research (9, 14). This strategy suffers from the classic tradeoff between precision and recall (i.e., to make more precise estimates at the cost of sensitivity). However, the decision threshold is hardly the only parameter that controls precision. C-statistics, widely used in model assessment, ceases to have meaning in such scenarios and the task shifts from binary classification to anomaly detection (i.e., sorting both needles and hay, rather than just needles) (15, 16). A better strategy for improving precision may be to evaluate the context in which models are developed or applied (e.g., training sample composition, observation period, or cohort characteristics) (1, 5, 10, 15, 17).

The variation in scale and scope of data used for training has a major influence on performance. A comprehensive meta-analysis noted that positive predictive values (PPV) corresponding to near-perfect sensitivity and specificity (both at 0.99) would be around 0.02, assuming a mortality rate of 20 per 100,000 patients, but PPV will increase to 0.33 for a mortality rate of 50 per 100,000. Indeed, the reported PPVs reveal a very strong concordance with suicide mortality ratio across 11 of the included studies (r = 0.971, p < 0.001), and no significant association between sensitivity and suicide mortality (r = 0.262, p = 0.437). In other words, precision is tied to the volume of non-events (i.e., no suicides), and with a larger control group there is less precision when classifying top 1^st^ and 5^th^ percentile of risk. Thus, non-events confound the appropriateness of most resampling methods to control for class imbalance (16, 18–20). Nonetheless, dropping the number of controls or training on extreme cases will lead to algorithmic bias and overfitting (21, 22).

Clinical characteristics that are strongly associated with suicide risk are also known to vary between cohorts and different time horizons (2, 7, 23–25). For example, a recent meta-analysis found suicide ideation to predict eventual death over 9 years to be roughly 0.3% of the time in non-psychiatric samples but 3.9% among psychiatric patients (2). This difference in precision mirrors sensitivities found for the same cohorts: 22% of suicide deaths were ascertained by ideation among non-psychiatric patients and 46% among psychiatric patients. Ironically, the odds ratio of death by suicide, given prior ideation, was roughly the same between groups (i.e., 3.86 for non-psychiatric and 3.23 for psychiatric patients), suggesting that the balance of cases-to-controls explains these differences in classification, and not the association of ideation and mortality.

Risk factors aside, it is important to consider denominator constraints that bias training data in a way that yields models that are poorly calibrated for a particular use case. As such, we propose evaluating the precision-recall tradeoff under different circumstances, using multiple samples of differing size and scope of features, but an identical cut point for classifying the response. Each model is then fitted using a cross-validation framework to parameterize uncertainty and illustrate trends for different rates of suicide during training, observation periods, and clinical subpopulations. We hypothesize that differential precision-recall can guide decisions about training data to maximize performance for anomaly detection, using a combination of sensitivity, PPV, F1 and area under the precision recall curve (AUPRC) as guides.

## Methods

### Participants and setting

A total of 1,649,577 patients from the Maryland Suicide Data Warehouse (MSDW) were selected for predictive modeling tasks. The MSDW is a statewide repository of deidentified patient-level health records, linked across hospital discharges (HSCRC), commercial claims (MHCC), and decedent information from the Maryland Office of the Chief Medical Examiner (OCME) (26–29). Decedents were selected on a database wide assessment of data quality, removing those with duplicative identification mappings, missing or invalid age/sex, missing or invalid Maryland Census tract or 5-digit ZIP code, and 1+ recorded encounter for either HSCRC or MHCC between January 1, 2016, and December 31, 2020.

Across the selected denominator of the MSDW, 1944 (0.1%) patients had been identified as suicide decedents who died between January 1, 2017, and December 31, 2020. Three control groups were identified by overlapping sources of information: 45,585 (2.8%) deceased patients from OCME who experienced a manner of death other than suicide, 847,716 (51.4%) living patients from HSCRC and 849,323 (51.5%) living patients from MHCC. There were 94,991 (5.8%) patients found in both the HSCRC and MHCC living control groups, which accounts for proportions not adding up to 100%. For living patients, a random index date was selected for the same outcome window as a proxy for date of death when determining windowed observations. The offset of January 1, 2016, to January 1, 2017, ensured at least 1 year of observation per living patient, even if no encounters occurred during that period.

### Feature definitions and selection

Several key predictive features were identified based on expert input and after reviewing relevant literature for suicide prediction modeling. Given the underlying data represented in MSDW, these features were limited to demographic, diagnostic, procedure, medication, and social need information. Diagnostic and procedure coded information included ICD-10-CM and CPT-4 coded observations categorized using the 2022 Clinical Classifications Software Refined (CCSR) tool (30–32) and the ‘comorbidity’ package of R, version 1.1.0 (33). Established pharmacy classes (EPC) were identified using MHCC data and the openFDA API look up tool for matching with the national drug code (NDC) (34). Social needs were identified using ICD-10-CM codes identified and validated in prior research (35). Geospatial clusters that correspond to regions in the state of Maryland, where suicide death is statistically elevated, were identified in earlier work and attached to these data by matching FIPS codes at the Census tract level for residence (36).

Only 7 of the 180 standalone attributes used in modeling, which correspond to demographics or patient level characteristics, did not change during the observation period (e.g., age at index date, region of last residence). All other variables were selected to reflect observations up to 7 days before the patient’s index date (i.e., date of death for decedents, and a random date for living patients). This is analogous to a health system only having recorded information up to the moment of assessment, even if some time has elapsed since the patient had their most recent encounter. Healthcare utilization was reflected by the number of encounters at certain points of care and within 30 or 365 days of the index date. Points of care included emergency room encounters, all cause hospitalization, outpatient or ambulatory care, and psychiatric hospitalization. Specific details on the selection and cleaning of our data can be obtained from the project GitHub repository (https://github.com/ckitche2/MSDW).

Several variables were combined as interaction terms for our models to control moderating effects. As controlling all interactions was not feasible, we selected a small number of conditions known to have a strong association with suicide risk that could be combined with other observations (1, 10, 25, 37). These interactions included CCSR primary categories for depressive, anxiety, bipolar and psychotic disorders, post-traumatic stress, attention deficit disorder, alcohol and opiate use disorders and prior ICD-10-CM coded observations for suicide ideation, attempt or intentional self-poisoning (1, 10, 25, 30, 37). Missingness was generally handled through zero imputation of binary features, but complete missingness in several key factors required development of source-specific feature sets (38, 39). Feature selection consisted of a single stage LASSO filter of the full decedent denominator to reduce the number of colinear or highly associated predictors. This cohort was chosen as it was the only one with fully available clinical and geographic features for these tasks (described below).

### Predictive modeling tasks

The modeling task consisted of 5-fold cross validation across 5 iterations to produce more meaningful estimates of generalizable performance. In each fit, a logistic regression model for suicide death was used with three sets of inputs, one for each source of data. This approach was used to account for differences in available attributes between HSCRC and MHCC, the former lacking pharmacy-based observations and the latter lacking marital status or geographic characteristics mapped to Census tract of residence. Decedents had the highest number of total available attributes, as they were selected on the basis of being found in one or both records, while source-specific cohorts were required for HSCRC-only and MHCC-only analyses. Living controls from the MHCC record were not robustly linked to those in the HSCRC, and vice versa.

Model performance was evaluated using a combination of classification and response metrics, assuming a decision threshold probability of 0.5 across all cases to facilitate interpretation. This means that for each model to predict a case of suicide, it must correspond to a response value where that outcome is more likely than not. The actual likelihood of suicide, contrasted with the predicted likelihood of a model, is small at virtually any threshold for risk (1). This tends to bias classification towards greater precision with this threshold, where a very small number of observations are expected to reach a probability of greather-than-0.5, especially when class imbalance is most pronounced (1, 2, 14).

Cross-validated performance for area under receiver operating characteristic (AUROC), area under precision-recall curve (AUPRC), point-sensitivity, and positive predictive value (PPV) were recorded and aggregated across all iterations and folds and with respect to the contrast tested. Estimates were considered significantly different if the average value fell outside of the 95% confidence interval of a comparison distribution of that same score.

### Sensitivity analyses

Three different contrasts were evaluated for the modeling task and for each cohort: (1) Non-suicide decedents: suicide versus other manners of death, (2) HSCRC: suicide versus living controls represented in hospital discharges, and (3) MHCC: suicide versus living controls represented in commercial insurance claims. These contrasts assessed training sample balances, prospective outcome windows, and clinical cohort types/stratified samples.

Sample balance refers to the ratio of control group patients to suicide decedents for each data source, where a base ratio consists of the full sample in each source. These approximate rates of suicide were 4.1% (roughly 1 in 24) among decedents, 0.2% (1 in 436) in HSCRC and 0.2% (1 in 437) in MHCC. While these rates are not directly comparable to those observed at the level of the general population (i.e., approximately 14 per 100,000, or 1 in 7,143), these rates reflect a substantial imbalance much like what is seen in prior suicide risk modeling research. Indeed, prior studies have used a variety of population samples with one model using a sample of 1 suicide in 2,778 U.S. veterans but another model using a sample with 1 suicide in 13 high-risk patients with prior suicidal behavior (1, 40–43). Each training sample composition was used for fitting a model that was then validated against a test data set that had a normal case-control composition reflective of the overall sample (e.g., 1 in 24; 1 in 436; and 1 in 437). This mimics a situation where researchers train models using a convenience sample of screened at-risk patients or through down sampling controls and then applying the model to a real-world sample/setting.

Outcome windows were identified by using the difference between index date and date of most recent encounter, ignoring cases where the two are identical. A binary variable was created as the outcome of interest: suicide death within 7 days (16.7% of suicide deaths), 30 days (32.5%), 90 days (46.6%), 180 days (57.4%) and 365 days (70.2%). Only the outcome of interest was varied for these tasks, while the ratio of case-to-control was constant across the cross-validation process (**Supplementary Figure 1**).

Finally, clinical cohort types (i.e., stratified samples) were contrasted to document meaningful differences in performance based on clinical subgroupings for patients aged 65 or older, aged 17 or younger, having 1+ ICD-10-CM coded social need, having 1+ ICD-10-CM coded psychiatric condition (among those identified for interaction terms), 1+ ICD-10-CM coded Charlson comorbidity index (33), 1+ emergency room visit, 1+ all cause hospitalization, and 1+ psychiatric hospitalization during the observation period. For these strata, performances may differ both in terms of clinical characteristics and the relative ratios of case-to-control; however, these strata highlight how expected values change as a function of clinical use case.

## Results

Several patient characteristics were summarized across each of the predictive modeling tasks, including demographics and select diagnostic information used for establishing our interaction terms (**Table 1**). Compared with either randomly selected living control group, suicide decedents were somewhat older (mean age 49.7 years), considerably more likely to be male (76.9%), have 1 or more depressive disorder diagnoses (40.3%), bipolar disorder (11.9%), anxiety disorders (32.2%), psychotic disorders (8.1%), post-traumatic stress (13.3%), ADHD (7.5%), prior suicide ideation or attempt (18.2%), opiate use disorder (9.9%), or alcohol use disorder (19.6%). However, compared to decedents where manner of death is other than suicide, few of these comparisons were as considerable except for average age (64.7 years), male sex (59.3%), and prior suicide ideation or attempt (5.4%; **Table 1**).

**Table 1:** Specification of the population cohorts used for predictive modeling tasks, by status as suicide case.

Features	Non-suicide decedents (Control)	Living HSCRC (Control)	Living MHCC (Control)	Suicide decedents (Case)
Patients	45,585	847,716	849,323	1944
Mean age ± SD	64.780 ± 19.644	42.011 ± 23.537	40.209 ± 22.929	49.729 (19.537)
Female (%)	18,561 (40.7)	466,977 (55.1)	442,797 (52.1)	449 (23.1)
Male (%)	27,024 (59.3)	380,739 (44.9)	406,526 (47.9)	1,495 (76.9)
Married (%)	14,455 (31.7)	308,137 (36.3)	–	685 (35.2)
Divorced (%)	4,570 (10)	40,149 (4.7)	–	220 (11.3)
Separated (%)	1,137 (2.5)	9,051 (1.1)	–	94 (4.8)
Widowed (%)	7,929 (17.4)	42,276 (5)	–	135 (6.9)
Depressive Dx (%)	16,140 (35.4)	44,115 (5.2)	36,216 (4.3)	784 (40.3)
Bipolar Dx (%)	4,081 (9)	10,441 (1.2)	5,279 (0.6)	232 (11.9)
Anxiety Dx (%)	13,042 (28.6)	46,615 (5.5)	45,495 (5.4)	625 (32.2)
Psychotic Dx (%)	2,910 (6.4)	5,311 (0.6)	2,220 (0.3)	158 (8.1)
PTSD Dx (%) *	4,012 (8.8)	9,299 (1.1)	18,747 (2.2)	259 (13.3)
ADHD Dx (%) *	1,679 (3.7)	7,906 (0.9)	12,008 (1.4)	145 (7.5)
SI-SA (%) *	2,448 (5.4)	8,603 (1)	112 (0)	354 (18.2)
OUD Dx (%) *	6,410 (14.1)	7,458 (0.9)	2,535 (0.3)	192 (9.9)
AUD Dx (%) *	8,914 (19.6)	13,858 (1.6)	4,501 (0.5)	381 (19.6)

*Names for diagnostic groupings abbreviated as PTSD, post-traumatic stress disorder; ADHD, attention deficit hyperactivity disorder; SI-SA, suicide ideation or attempt; OUD, opiate use disorder; AUD, alcohol use disorder.

**Figure 1:**
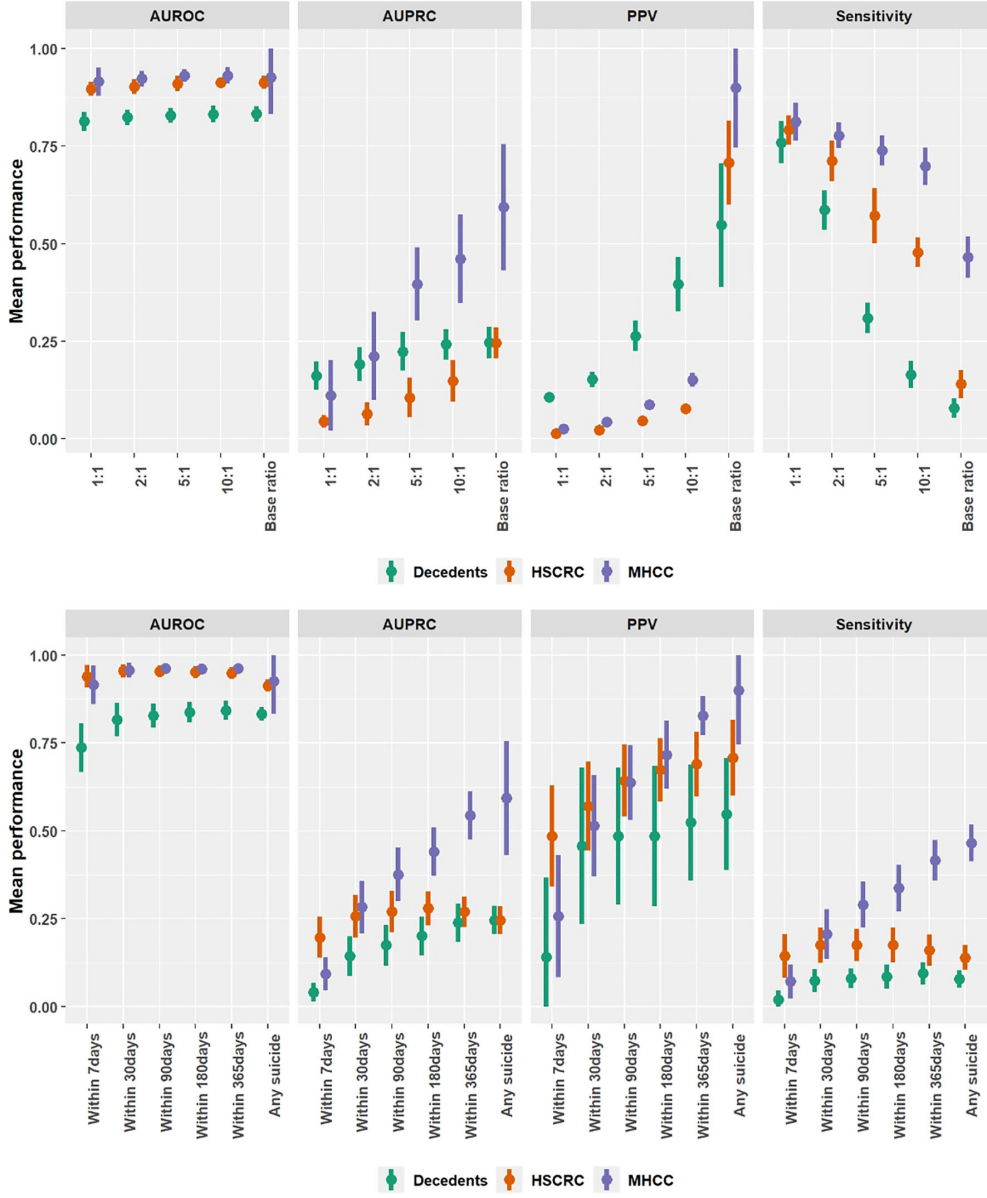
Mean and 95% confidence interval of predictive model performances contrasting suicide decedents with different control groups based on sampling ratios (top row) and time windows (bottom row).

The average estimated performance for each contrast in the training sample ratio and prospective time window tasks were measured using AUROC, AUPRC, PPV and sensitivity (**Table 2**) and further visualized with 95% confidence intervals (**Figure 1**). For both tasks (i.e., varying sampling ratios and time windows), AUROC did not change significantly from the initial fit (i.e., 1-to-1 training ratio and 7-day prospective window) to using the full control group denominator in training and prospective suicide prediction without a window. The one exception was for non-suicide decedents and only in the prospective time window task, where AUROC is improved by roughly 0.1 (i.e., AUROC increased from 0.737 to 0.832; **Table 2**).

**Table 2:** Average cross-validated performance of models based on sampling ratios and time windows when compared to a base model.

Contrast	Decedents				HSCRC				MHCC			
	AUROC	AUPRC	PPV	Sensitivity	AUROC	AUPRC	PPV	Sensitivity	AUROC	AUPRC	PPV	Sensitivity
Training sample ratio												
1 to 1 (base)	0.813	0.162	0.106	0.760	0.897	0.045	0.014	0.791	0.915	0.111	0.025	0.813
1 to 2	0.824	0.191	0.152*	0.586*	0.903	0.064*	0.022*	0.711*	0.923	0.212*	0.043*	0.777
1 to 5	0.829	0.223*	0.264*	0.309*	0.910	0.106*	0.045*	0.571*	0.930	0.396*	0.087*	0.739*
1 to 10	0.832	0.242*	0.396*	0.165*	0.912	0.148*	0.076*	0.478*	0.931	0.461*	0.151*	0.698*
Full denominator	0.832	0.246*	0.548*	0.078*	0.913	0.246*	0.708*	0.140*	0.927	0.593*	0.899*	0.465*
Prospective time window												
7 days (base)	0.737	0.040	0.141	0.018	0.939	0.197	0.485	0.144	0.916	0.093	0.257	0.071
30 days	0.816*	0.143*	0.457*	0.073*	0.955	0.256*	0.570	0.174	0.957	0.282*	0.514*	0.206*
90 days	0.828*	0.175*	0.485*	0.080*	0.954	0.270*	0.643*	0.175	0.962	0.376*	0.638*	0.290*
180 days	0.838*	0.201*	0.485*	0.085*	0.952	0.280*	0.673*	0.175	0.961	0.441*	0.716*	0.337*
365 days	0.843*	0.238*	0.524*	0.094*	0.949	0.269*	0.689*	0.160	0.962	0.544*	0.827*	0.416*
Any suicide	0.832*	0.246*	0.548*	0.078*	0.913	0.246	0.708*	0.140	0.927	0.593*	0.899*	0.465*

Contrasts reflect separate cohorts defined through source of data and control group, for decedents of both sources, suicide decedents and living patients of the Health Services Cost Review Commission (HSCRC) and Maryland Health Care Commission (MHCC).

*Average point-estimated performance is significantly different from base at p <.05.

AUPRC is significantly improved for all data sources moving towards our full denominator in training and any prospective suicide. For non-suicide decedents, AUPRC shifts from 0.162 when the training composition is 1-to-1 to 0.246 for the full denominator. AUPRC shifts from 0.045 to 0.246, and from 0.111 to 0.593, for living controls of HSCRC and MHCC, accordingly. While overall AUPRC is increasing, PPV (precision) increases with the progression while sensitivity decreases, but at different rates for each data source (**Table 2**).

AUPRC, PPV, and sensitivity tended to increase uniformly with window size, suggesting no precision-recall trade-off exists for these horizons. For time windows of both non-suicide decedents and HSCRC’s living controls, a slight tendency was observed for point-sensitivity to peak between 6 and 12 months before declining for any prospective suicide. This reduced sensitivity corresponds to the addition of suicide cases that have a gap greater than 12 months between last encounter and date of death and might therefore reflect patients for whom we have insufficient clinical information or no routine care. Indeed, roughly 34.5% of suicide decedents had dates of death beyond 12 months from their last clinical encounter (**Supplementary Table 1**).

**Figure 2:**
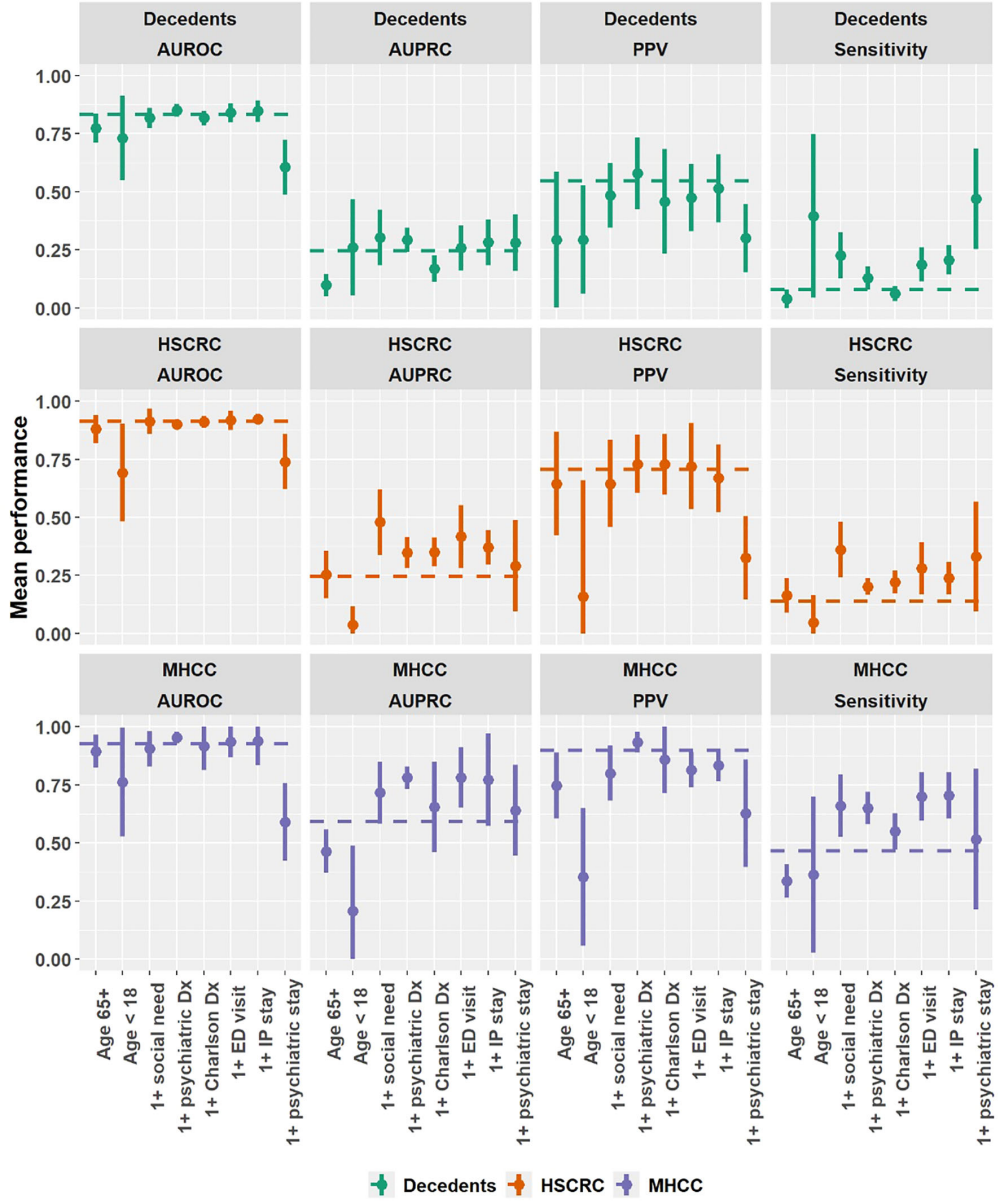
Mean and 95% confidence interval of predictive model performances contrasting suicide decedents with different control groups based on select stratified samples*. * Horizontal dashed line for each trace represents performances for the full cohort in each data source.

Performances of suicide prediction models for stratified samples were also measured along with full denominator counts and respective suicide rates (**Table 3**). Model performances, along with their respective 95% confidence intervals, were also visualized for each data source (**Figure 2**). AUROC was not significantly different from the full cohort estimates across all three data sources, except for patients with a history of psychiatric hospitalization where it was significantly lower (non-suicide decedents: 0.606; living HSCRC: 0.740; and living MHCC: 0.509). Considerable variability was observed in AUROC for both psychiatric patients and those aged younger than 18 years (**Table 3**).

**Table 3:** Performance of models contrasting stratified samples by source of data.

Cohort	Decedents						HSCRC						MHCC					
	Count (%)	Rate (100K)	AUROC	AUPRC	PPV	Sensitivity	Count (%)	Rate (100K)	AUROC	AUPRC	PPV	Sensitivity	Count (%)	Rate (100K)	AUROC	AUPRC	PPV	Sensitivity
Total	47,529 (100)	4,090	0.832	0.246	0.548	0.078	849,660 (100)	229	0.913	0.246	0.708	0.140	851,267 (100)	228	0.927	0.593	0.899	0.465
Age 65+	24,588 (51.7)	1,842	0.773*	0.098*	0.294*	0.039*	167,819 (19.8)	270	0.88*	0.254	0.645	0.164	139,037 (16.3)	326	0.895	0.464	0.747	0.336*
Age < 18	704 (1.5)	9,943	0.731*	0.260	0.294*	0.396*	150,251 (17.7)	47	0.692*	0.036*	0.160*	0.047*	172,013 (20.2)	41	0.762*	0.208*	0.353*	0.364*
1+ Charlson Dx	36,140 (76)	2,507	0.818	0.169*	0.458	0.062	156,736 (18.4)	578	0.911	0.351*	0.728	0.221*	104,855 (12.3)	864	0.917	0.655	0.858	0.549*
1+ psych Dx	32,577 (68.5)	3,855	0.851	0.293*	0.579	0.129*	132,685 (15.6)	947	0.900	0.349*	0.730	0.203*	89,399 (10.5)	1,405	0.953	0.781*	0.934	0.649*
1+ social need	7,651 (16.1)	4,117	0.817	0.303*	0.485	0.226*	17,068 (2)	1,846	0.913	0.479*	0.645	0.361*	24,847 (2.9)	1,268	0.905	0.717	0.800	0.660*
1+ ED visit	30,579 (64.3)	2,600	0.840	0.259	0.474	0.187*	70,701 (8.3)	1,124	0.918	0.417*	0.720	0.281*	26,092 (3.1)	3,047	0.937	0.782*	0.815	0.699*
1+ IP stay	32,866 (69.1)	2,775	0.848	0.282	0.515	0.206*	111,391 (13.1)	819	0.924	0.371*	0.669	0.239*	34,055 (4)	2,678	0.938	0.773*	0.834	0.705*
1+ psych stay	1,721 (3.6)	11,621	0.606*	0.280	0.302*	0.469*	3186 (0.4)	6,277	0.740*	0.290*	0.326*	0.331*	275 (0)	72,727	0.590*	0.641	0.628*	0.516
Pearson’s r			-0.604	0.362	-0.375	0.828**			0.524	0.867**	0.430	0.938**			0.563	0.759**	0.391	0.869**

Rate of suicide death per capita reflects proportion of patients who died by suicide relative to substrata sample size, rather than a population estimate and significance testing performed within metrics to determine meaningful difference with full sample performance estimates (e.g., first row). Pearson’s r reflects strength of correlation for each metric with rate of suicide death, excluding psychiatric hospitalizations, which were exceedingly rare across data source.

*Average point-estimated performance is significantly different from total sample performance at p <.05.

**Statistically significant statistic at p <.05.

Compared with total cohort performance, patients with 1 or more psychiatric conditions were found to have significantly higher AUPRC (non-suicide decedents: 0.293; living HSCRC: 0.349; and living MHCC: 0.781) and point-sensitivities (0.129, 0.203, and 0.649), but not point-precision (0.579, 0.730, and 0.934). Notably, the rates of suicide death increased for both HSCRC (229/100K, to 947/100K) and MHCC (228/100K, to 1405/100K), but changed minimally for non-suicide decedents (4090/100K, to 3855/100K). A similar case was observed for patients with 1 or more ICD-10-CM coded social need. AUPRC and point-sensitivities were significantly higher compared to total cohort for decedents (0.303 and 0.226, respectively), living HSCRC (0.479 and 0.361), and just point-sensitivity for living MHCC (0.660; **Table 3**).

Overall, AUPRC was greater than that of the total sample for patients with social needs, psychiatric conditions, emergency visits and all cause hospital stays and only for the data sources using living controls (**Table 3**). Wherever AUPRC was significantly higher, point-sensitivity was higher as well, and PPV was generally lower or at level with total sample performance (**Figure 2**). Though only 8 samples were evaluated using this approach (after excluding rare psychiatric hospitalizations), AUPRC was not significantly associated with rate of suicide, except where models were derived from MHCC records (r = 0.759, p < 0.05). In all three sources of data, point-estimated sensitivity was strongly and positively correlated with rate of suicide (decedents: r = 0.828, p < 0.05; HSCRC: r = 0.938, p < 0.05; MHCC: r = 0.869, p < 0.05), but PPV was not. Rate of suicide death was uniformly the greatest among patients with a psychiatric hospitalization, however, making it an outlier for these correlations, and thus excluded from the aforementioned statistics.

## Discussion

Predicting suicide death is often a challenging task due to the extreme class imbalance between suicide deaths versus the rest of the population (1–3). The low PPV of the suicide death prediction models often result in large false positives being identified as suicidal, thus limiting the generalizability as well as cost-effectiveness of such models in clinical settings (1, 4). To address these issues, this study evaluated the precision-recall tradeoff under different circumstances, using multiple samples of differing size and scope of features, but an identical cut point for classifying the response. Each model was fitted using a cross-validation framework to parameterize uncertainty and illustrate trends for different rates of suicide during training, observation periods, and clinical subpopulations.

Several findings were notable in this study. First, the practice of systematically undersampling control groups to account for substantial imbalance in real-world suicide events should be viewed with caution (1, 44). Many researchers using machine learning algorithms encounter this concern but then fail to consider trade-offs in precision and recall, noting instead that the AUROC does not change much by undersampling. Negatively biased AUPRC were observed in all three data sources as a consequence of undersampling and progressively worsened precision (**Tables 2**, **3**). Risk estimates obtained this way will tend to overstate true risk, leading to many false positives and poor application in decision support (e.g., alert fatigue).

Second, and conversely, relying on imbalanced samples for training and test cases favored point-precision at the cost of missed events. Furthermore, the progressive decay in sensitivity was very different for each of the three sources of data (**Table 2**). For example, compared to the decedents and HSCRC, estimates in the MHCC had the shallowest decay for sensitivity. Such a difference can be attributed to both the large number of living controls in MHCC as well as the availability of pharmacy records with other clinically relevant information (e.g., accounting for an interaction between medication and psychiatric condition among living individuals). In other words, it is possible for predictions to achieve enhanced precision and sensitivity simultaneously when using an appropriate imbalance between cases and control groups as well as the availability of more comprehensive clinical attributes that are predictive of suicide.

Third, temporal proximity to date of death and availability of clinical information can affect precision-recall performance. The more temporally distant death is from the last encounter, the better the AUPRC and point-precision of estimates (**Table 2**). However, when all suicide deaths, including those outside of 12-months are considered, the improvements plateau or reverse. We hypothesize that the roughly 34% of suicide decedents that have no encounters within a year of their death likely represent a portion of the cohort with poor healthcare access and very low utility (**Supplementary Table 1**). This calls into question the completeness of their clinical information and appropriateness for inclusion in the prediction task. After all, if a decedent’s record only consisted of a single encounter for an urgent care visit, we can hardly expect much information by way of mental health screening or treatment. These information gaps are attributable to data quality or missingness and a challenge for statistical risk assessment (45).

Finally, all else being equal, the clinical characteristics of a population can substantially affect model performance, making it a challenge in the first place to identify a one-size-fits-all risk model. AUPRC is significantly improved by subsetting each data source by patients having one or more psychiatric diagnosis (**Table 3**). This is hardly surprising since the rate of suicide death is higher among psychiatric patients (and therefore a lower imbalance of outcome), but sensitivity appears to be improved more than precision, mirroring the same trend as sample training imbalance (i.e., more balance, more sensitivity). Those with social needs, emergency room visitation, or hospitalizations seem to fit this pattern in HSCRC and MHCC cohorts as well (**Table 3**).

Our study findings are largely consistent with those of a meta-analysis conducted by Belsher et al, and observations by others (1, 2, 10, 11). Cross-validated precision is improved by increasing the classification threshold for risk (e.g., 95^th^ to 99^th^ percentile of risk), but is also tied to control group size. By increasing class imbalance in data during training and thereby decreasing the rate of suicide, our model has learned a response that yields better PPV in test data. The reverse is also true in our analysis, in which sensitivity is improved by lowering the classification threshold (e.g., 99^th^ to 95^th^ percentile of risk) and decreasing control group imbalance (i.e., increased rate of suicide). The main difference between our experiment and Belsher’s meta-analysis is that we used a static classification threshold for all analyses (i.e., the response likelihood falling above 0.5). To our knowledge, this analysis is the only endeavor to identify a trend in AUPRC improving with increasing imbalance in suicide mortality, either through modifying training sample imbalance, changing the outcome horizon, or selecting a clinical substratum for model development.

The findings of this study can be used to increase the generalizability of suicide prediction models; however, this study has a few limitations. First, although the data used in this study (i.e., MSDW) is considered the gold standard in identifying suicide cases in Maryland, a considerable number of deaths are categorized as ‘undetermined’ by the OCME even when suicide is suspected in post-mortem findings. Our prior work has suggested that a disproportionate number of these cases affect minority populations and those with limited access to care, thus our results may not show the true effect of class imbalance in such populations (46, 47). Additionally, the MHCC data was also limited to commercial claim records only thus potentially biasing our sample towards higher income working age individuals.

Thus, our findings might require replication in an external, nationally representative source of data. Second, we identified the list of features used in our prediction models using expert feedback and a review of literature; however, a more systematic review of the literature may result in a different set of predictors. Future research should explore the effect of other data types, especially ones not represented in our data (e.g., lab results), on the effect of class imbalance on predicting suicide. Furthermore, administrative claims and discharge records lack any characterization of symptom severity, toxicology findings, screening, assessment, or clinical narratives. We plan to both leverage and validate electronic health records containing such relevant predictors in our future work. Third, we used a regression model to assess the effect of sampling, time windows, and population strata on the performance and generalizability of the models. Future research should test these effects on non-parametric and ensemble AI algorithms, and how fine tuning such models will affect the outcomes of interest. We conducted a parallel set of analyses using an extreme gradient boosted tree, and found very similar findings, especially incremental improvement in AUPRC (**Supplementary Table 2**). This suggests these findings of class imbalance translate to other families of algorithms. Finally, our study only focused on model generalizability and performance as it relates to the class imbalance challenges and did not consider its potential effect on cost-effectiveness of such models in practice (48). Indeed, the balance of costs has not yet been explored thoroughly in our work but might be documented by varying the weight of recall relative to precision as a series of F-beta statistics to support stakeholder decisions on whether to adopt a statistical model (49, 50). When there is a clear idea of costs associated with type 1 and type 2 errors, the same model can be viewed as working well for some clinical populations over others. Put differently, the tolerance for misclassifying cases of suicide will depend on what kinds of interventions and routine care are involved, as well as size of cohort and timing of risk.

## Conclusion

Prediction of rare events, like death by suicide, in real-world observational data is challenging as the probability of the event is far less likely than observing non-events at every level of risk. In a series of experiments, we have demonstrated that AUPRC is improved through training models under the same imbalance as the eventual use case (i.e., test data), and through maximizing the time horizon for which we hoped to predict outcomes. A careful selection of training samples is required for enhanced model precision. Higher cohort-specific rates of death (i.e., greater class balance) tended to result in highly sensitive performance, but often at the cost of lower average precision. Finally, the precision and recall of fitted models is affected by idiosyncratic risks, such that different points of care or patient populations might benefit from specially tuned models to fit their use case.

## Data Availability

The datasets presented in this article are not readily available because previously established agreements for data use and access with respective custodians and providers prohibit use and sharing of these data beyond the scope of our original research. Requests to access the datasets should be directed to ckitche2@jh.edu.
