# Supplementary figures and images for: Navigating extreme class imbalance in suicide risk prediction
Source: Front Psychiatry. 2026 Jan 12;16:1679618. doi: 10.3389/fpsyt.2025.1679618 (PMC12833411; doi:10.3389/fpsyt.2025.1679618)

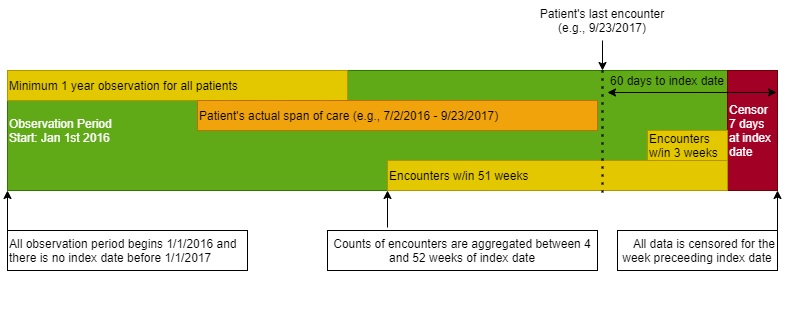

Supplement: Supplementary file 1 [file Image1.jpeg]
