# Supplementary material for: Navigating extreme class imbalance in suicide risk prediction
Source: Front Psychiatry. 2026 Jan 12;16:1679618. doi: 10.3389/fpsyt.2025.1679618 (PMC12833411; doi:10.3389/fpsyt.2025.1679618)
Supplement: Supplementary file 2 [file Table1.docx]

**SUPPLEMENTAL MATERIALS**

**Table S1**. Characteristics of suicide decedents whose encounters are only available prior to 12 months, 1 month and 7 days before death.

| **Scope** |  | **Only encounters before** | | | **Only encounters within** |
| --- | --- | --- | --- | --- | --- |
| **Feature** | **All suicide decedents** | **12mos** | **1mos** | **7days** | **7days** |
| Total | 1,944 | 670 | 1,492 | 1,690 | 254 |
| Mean age ± SD | 49.7 ± 19.5 | 46.4 ± 18.6 | 47.9 ± 18.9 | 48.9 ± 19.1 | 55.0 ± 21.5 |
| Female (%) | 449 (23.1) | 108 (16.1) | 324 (21.7) | 380 (22.5) | 69 (27.2) |
| Male (%) | 1495 (76.9) | 562 (83.9) | 1168 (78.3) | 1310 (77.5) | 185 (72.8) |
| Married (%) | 685 (35.2) | 214 (31.9) | 502 (33.6) | 591 (35) | 94 (37) |
| Divorced (%) | 220 (11.3) | 69 (10.3) | 167 (11.2) | 193 (11.4) | 27 (10.6) |
| Separated (%) | 94 (4.8) | 22 (3.3) | 71 (4.8) | 83 (4.9) | 11 (4.3) |
| Widowed (%) | 135 (6.9) | 31 (4.6) | 83 (5.6) | 104 (6.2) | 31 (12.2) |
| Depressive Dx (%) | 784 (40.3) | 125 (18.7) | 509 (34.1) | 642 (38) | 142 (55.9) |
| Bipolar Dx (%) | 232 (11.9) | 25 (3.7) | 143 (9.6) | 195 (11.5) | 37 (14.6) |
| Anxiety Dx (%) | 625 (32.2) | 80 (11.9) | 388 (26) | 501 (29.6) | 124 (48.8) |
| Psychotic Dx (%) | 158 (8.1) | 22 (3.3) | 105 (7) | 127 (7.5) | 31 (12.2) |
| PTSD Dx (%) | 259 (13.3) | 38 (5.7) | 161 (10.8) | 210 (12.4) | 49 (19.3) |
| ADHD Dx (%) | 145 (7.5) | 32 (4.8) | 97 (6.5) | 122 (7.2) | 23 (9.1) |
| SI-SA (%) | 354 (18.2) | 40 (6) | 228 (15.3) | 299 (17.7) | 55 (21.7) |
| OUD Dx (%) | 192 (9.9) | 31 (4.6) | 130 (8.7) | 151 (8.9) | 41 (16.1) |
| AUD Dx (%) | 381 (19.6) | 67 (10) | 267 (17.9) | 325 (19.2) | 56 (22) |

Names for diagnostic groupings abbreviated as PTSD (post-traumatic stress disorder), ADHD (attention deficit hyperactivity disorder), SI-SA (suicide ideation or attempt), OUD (opiate use disorder) and AUD (alcohol use disorder).

**Table S2.** Average model performance across multiple sampling and time window contrasts, using XGBoost algorithm.

|  | **Decedents** | | | | **HSCRC** | | | | **MHCC** | | | |
| --- | --- | --- | --- | --- | --- | --- | --- | --- | --- | --- | --- | --- |
| *Contrast* | *AUROC* | *AUPRC* | *PPV* | *Sensitivity* | *AUROC* | *AUPRC* | *PPV* | *Sensitivity* | *AUROC* | *AUPRC* | *PPV* | *Sensitivity* |
| Training sample ratio | | | | | | | | | | | | |
| 1 to 1 (base) | 0.832 | 0.219 | 0.109 | 0.778 | 0.942 | 0.209 | 0.017 | 0.863 | 0.923 | 0.245 | 0.014 | 0.847 |
| 1 to 2 | 0.838 | 0.230 | 0.161* | 0.588* | 0.947 | 0.265 | 0.029* | 0.813* | 0.925 | 0.315 | 0.025* | 0.789* |
| 1 to 5 | 0.839 | 0.239 | 0.280* | 0.303* | 0.950 | 0.341* | 0.064* | 0.739* | 0.924 | 0.392* | 0.058* | 0.723* |
| 1 to 10 | 0.840 | 0.251 | 0.429* | 0.153* | 0.954 | 0.404* | 0.124* | 0.685* | 0.917 | 0.437* | 0.101* | 0.668* |
| Full denominator | 0.841 | 0.255* | 0.583* | 0.065* | 0.948 | 0.666* | 0.937* | 0.507* | 0.885* | 0.560* | 0.856* | 0.475* |
| Prospective time window | | | | | | | | | | | | |
| 7 days (base) | 0.867 | 0.163 | 0.993 | 0.102 | 0.935 | 0.277 | 0.999 | 0.308 | 0.898 | 0.261 | 0.999 | 0.314 |
| 30 days | 0.859 | 0.191 | 0.987* | 0.121 | 0.944 | 0.284 | 0.999 | 0.313 | 0.912 | 0.319 | 0.999 | 0.374 |
| 90 days | 0.853 | 0.202* | 0.981 | 0.119 | 0.945 | 0.348* | 0.999 | 0.375* | 0.906 | 0.346* | 0.999 | 0.415* |
| 180 days | 0.858 | 0.230* | 0.977* | 0.128 | 0.955* | 0.444* | 0.999 | 0.446* | 0.908 | 0.409* | 0.999 | 0.475* |
| 365 days | 0.863* | 0.261* | 0.972* | 0.146* | 0.962* | 0.538* | 0.999 | 0.526* | 0.908 | 0.521* | 0.999 | 0.584* |
| Any suicide | 0.841* | 0.255* | 0.960* | 0.177 | 0.948 | 0.666* | 0.999* | 0.658* | 0.885 | 0.560* | 0.999 | 0.611* |

Contrasts reflect separate cohorts defined through source of data and control group, for decedents of both sources, suicide decedents and living patients of the Health Services Cost Review Commission (HSCRC) and Maryland Health Care Commission (MHCC).
* Average point-estimated performance is significantly different from base statistically significant at p < 0.05

**Table S3.** Feature sets for each source of data after selection with LASSO to reduce colinearity.

| **Variable** | **Definition** | **Decedent** | **HSCRC** | **MHCC** |
| --- | --- | --- | --- | --- |
| nd_adhd_7days_AND_counseling_med_eval_7days | encounter with ADHD and counseling/medication evaluation up to 7 days of index | X | X | X |
| nd_adhd_7days_AND_NRI_7days | encounter with ADHD and claim for NRI up to 7 days of index | X |  | X |
| si_sa_7days | encounter with suicide ideation or attempt up to 7 days of index | X | X | X |
| psychotic_7days_AND_psych_ip_1mos | encounter with psychotic illness up to 7 days and psychiatric hospitalization within 1 month of index | X | X | X |
| ptsd_7days_AND_counseling_7days | encounter with PTSD and counseling service up to 7 days of index | X | X | X |
| anxiety_7days_AND_NRI_7days | encounter with anxiety and claim for NRI up to 7 days of index | X |  | X |
| psychotic_7days_AND_counseling_7days | encounter with psychotic illness and counseling service up to 7 days of index | X | X | X |
| eating_7days | encounter with eating disorder up to 7 days of index | X | X | X |
| nd_adhd_7days_AND_ED_1mos | encounter with ADHD up to 7 days and ED visit within 1 month of index | X | X | X |
| dementia_7days | encounter with dementia up to 7 days of index | X | X | X |
| male | male sex | X | X | X |
| psych_ip_12mos | psychiatric hospitalization within 12 months of index | X | X | X |
| psych_ip_7days | psychiatric hospitalization up to 7 days of index | X | X | X |
| si_sa_7days_AND_counseling_7days | encounter with suicide ideation or attempt and counseling up to 7 days of index | X | X | X |
| count_psych_ip_within_7days | count of psychiatric hospitalizations up to 7 days of index | X | X | X |
| nd_adhd_7days_AND_typical_antipsych_7days | encounter with ADHD and use of typical antipsychotic up to 7 days of index | X |  | X |
| opiates_7days_AND_atypical_antipsych_7days | encounter with opiate use disorder and use of atypical antipsychotic up to 7 days of index | X |  | X |
| opiates_7days_AND_counseling_med_eval_7days | encounter with opiate use disorder and counseling with medical evaluation up to 7 days of index | X | X | X |
| ptsd_7days_AND_atypical_antipsych_7days | encounter with PTSD and use of atypical antipsychotic up to 7 days of index | X |  | X |
| alcohol_7days_AND_pharma_management_7days | encounter with alcohol use disorder and psychiatric pharmacology management evaluation up to 7 days of index | X | X | X |
| depressive_7days_AND_typical_antipsych_7days | encounter with depressive disorder and use of typical antipsychotic up to 7 days of index | X |  | X |
| ptsd_7days_AND_partial_opi_agonist_7days | encounter with PTSD and use of partial opiate agonist up to 7 days of index | X |  | X |
| ptsd_7days_AND_counseling_med_eval_7days | encounter with PTSD and counseling with medical evaluation up to 7 days of index | X | X | X |
| nd_adhd_7days_AND_SNRI_7days | encounter with ADHD and use of SNRI up to 7 days of index | X |  | X |
| hx_self_harm_7days | history of self harm up to 7 days of index | X | X | X |
| pharma_management_7days | encounter with psychiatric pharmacology management evaluation up to 7 days of index | X | X | X |
| si_sa_7days_AND_opi_antagonist_7days | encounter with suicide ideation or attempt and use of opiate antagonist up to 7 days of index | X |  | X |
| mood_stabilizer_7days | use of mood stabilizer up to 7 days of index | X |  | X |
| chf_7days | encounter with congestive heart failure up to 7 days of index | X | X | X |
| si_sa_7days_AND_IP_1mos | encounter with suicide ideation or attempt and inpatient up to 7 days of index | X | X | X |
| nd_adhd_7days_AND_psych_ip_1mos | encounter with ADHD up to 7 days and psychiatric hospitalization within 1 month of index | X | X | X |
| bh_sleep_7days | encounter with sleep disorder up to 7 days of index | X | X | X |
| bipolar_7days_AND_family_therapy_7days | encounter with bipolar and family counseling or therapy up to 7 days of index | X | X | X |
| depressive_7days | encounter with depressive disorder up to 7 days of index | X | X | X |
| bipolar_7days_AND_counseling_7days | encounter with bipolar and any counseling services up to 7 days of index | X | X | X |
| bipolar_7days_AND_gaba_a_agonist_7days | encounter with bipolar and use of GABA A agonist up to 7 days of index | X |  | X |
| alcohol_7days_AND_psych_ip_1mos | encounter with alcohol use disorder up to 7 days and psychiatric hospitalization within 1 month of index | X | X | X |
| si_sa_7days_AND_IP_12mos | encounter with suicide ideation or attempt up to 7 days and inpatient within 12 months of index | X | X | X |
| hepatitis_7days | encounter with hepatitis up to 7 days of index | X | X | X |
| alcohol_7days_AND_psych_interview_exam_7days | encounter with alcohol use disorder and psychiatric interview or exam up to 7 days of index | X | X | X |
| bipolar_7days_AND_pharma_management_7days | encounter with bipolar and psychiatric pharmacology management evaluation up to 7 days of index | X | X | X |
| opiates_7days_AND_cns_stimulant_7days | encounter with opiate use disorder and use of a CNS stimulant up to 7 days of index | X |  | X |
| obesity_7days | encounter with obesity up to 7 days of index | X | X | X |
| psych_interview_exam_7days | encounter with psychiatric interview or exam up to 7 days of index | X | X | X |
| anxiety_7days_AND_pharma_management_7days | encounter with anxiety disorder and psychiatric pharmacological management evaluation up to 7 days of index | X | X | X |
| immigration_issues_7days | encounter with identified immigration issues up to 7 days of index | X | X | X |
| opiates_7days_AND_ED_eval_any_severity_7days | encounter with opiate use disorder and CPT-coded ED evaluation of any severity up to 7 days of index | X | X | X |
| depressive_7days_AND_anti_epileptic_7days | encounter with depressive disorder and use of antiepileptic up to 7 days of index | X |  | X |
| anxiety_7days_AND_counseling_med_eval_7days | encounter with anxiety disorder and counseling with medical evaluation up to 7 days of index | X | X | X |
| si_sa_7days_AND_psych_ip_7days | encounter with suicide ideation or attempt and psychiatric hospitalization up to 7 days of index | X | X | X |
| stimulant_7days | encounter with stimulant use disorder up to 7 days of index | X | X | X |
| psychotic_7days_AND_psych_ip_12mos | encounter with psychotic illness up to 7 days and psychiatric hospitalization within 12 months of index | X | X | X |
| IP_12mos | encounter with inpatient hospitalization within 12 months of index | X | X | X |
| tobacco_7days | encounter with tobacco use disorder up to 7 days of index | X | X | X |
| opiates_7days_AND_psych_ip_12mos | encounter with opiate use disorder up to 7 days and psychiatric hospitalization within 12 months of index | X | X | X |
| bipolar_7days_AND_anticholinergic_7days | encounter with bipolar and use of anticholinergic up to 7 days of index | X |  | X |
| SSRI_7days | use of SSRI up to 7 days of index | X |  | X |
| opiates_7days_AND_male | encounter with opiate use disorder up to 7 days of index and male sex | X | X | X |
| bipolar_7days_AND_IP_1mos | encounter with bipolar up to 7 days of index and hospitalization within 1 month of index | X | X | X |
| si_sa_7days_AND_psychotherapy_or_addon_7days | encounter with suicide ideation or attempt and psychotherapy with or without psychotherapy add on up to 7 days of index | X | X | X |
| bipolar_7days_AND_benzo_hypn_anxio_7days | encounter with bipolar and use of benzo/hypnotic/anxiolytic up to 7 days of index | X |  | X |
| benzo_hypn_anxio_7days | use of benzo/hypnotic/anxiolytic up to 7 days of index | X |  | X |
| psychotic_7days_AND_benzo_hypn_anxio_7days | encounter with psychotic illness and use of benzo/hypnotic/anxiolytic up to 7 days of index | X |  | X |
| OP_7days | encounter with outpatient care up to 7 days of index | X | X | X |
| diabetes_comp_7days | encounter with complications of diabetes up to 7 days of index | X | X | X |
| pulmonary_dis_7days | encounter with pulmonary disease up to 7 days of index | X | X | X |
| si_sa_7days_AND_psych_interview_exam_7days | encounter with suicide ideation or attempt and psychiatric interview or exam up to 7 days of index | X | X | X |
| other_substance_7days | encounter with other substance use disorder up to 7 days of index | X | X | X |
| psychotic_7days_AND_other_psych_px_7days | encounter with psychotic illness and other psychiatric care procedure up to 7 days of index | X | X | X |
| alcohol_7days_AND_psych_ip_12mos | encounter with alcohol use disorder up to 7 days and psychiatric hospitalization within 12 months of index | X | X | X |
| bipolar_7days_AND_psych_ip_1mos | encounter with bipolar up to 7 days and psychiatric hospitalization within 1 month of index | X | X | X |
| bipolar_7days_AND_mood_stabilizer_7days | encounter with bipolar and use of mood stabilizer up to 7 days of index | X |  | X |
| IP_7days | encounter with inpatient hospitalization up to 7 days of index | X | X | X |
| psychotic_7days_AND_EM_99204_99215_4_7days | encounter with psychotic illness and EM codes 99204 or 99215 up to 7 days of index | X | X | X |
| essential_htn_7days | encounter with essential hypertension up to 7 days of index | X | X | X |
| renal_dis_7days | encounter with renal disease up to 7 days of index | X | X | X |
| ptsd_7days_AND_anti_epileptic_7days | encounter with PTSD and use of antiepileptic up to 7 days of index | X |  | X |
| opiates_7days_AND_benzo_hypn_anxio_7days | encounter with opiate use disorder and use of benzo/hypnotic or anxiolytic up to 7 days of index | X |  | X |
| neurodevelop_7days | encounter with neurodevelopmental disorder up to 7 days of index | X | X | X |
| ptsd_7days_AND_opi_agonist_7days | encounter with PTSD and use of opiate agonist up to 7 days of index | X |  | X |
| opiates_7days_AND_opi_agonist_7days | encounter with opiate use disorder and use of opiate agonist up to 7 days of index | X |  | X |
| EM_99214_3_7days | encounter with EM code 99214 up to 7 days of index | X | X | X |
| si_sa_7days_AND_male | encounter with suicide ideation or attempt up to 7 days of index and male sex | X | X | X |
| ptsd_7days_AND_NRI_7days | encounter with PTSD and use of NRI up to 7 days of index | X |  | X |
| other_psych_px_7days | encounter with other psychiatric procedure codes up to 7 days of index | X | X | X |
| pvd_7days | encounter with peripheral vascular disease up to 7 days of index | X | X | X |
| cns_stimulant_7days | encounter with use of CNS stimulant up to 7 days of index | X |  | X |
| hx_abuse_neglect_7days | history of abuse of neglect up to 7 days of index | X | X | X |
| personality_7days | encounter with personality disorder up to 7 days of index | X | X | X |
| psychotic_7days_AND_pharma_management_7days | encounter with psychotic illness and psychiatric pharmacology management up to 7 days of index | X | X | X |
| psychotherapy_or_addon_7days | encounter with psychotherapy or psychotherapy add on code up to 7 days of index | X | X | X |
| rend_7days | encounter with CCI-defined renal disease up to 7 days of index | X | X | X |
| diabetes_uncomp_7days | encounter with diabetes without complications up to 7 days of index | X | X | X |
| alcohol_7days_AND_SNRI_7days | encounter with alcohol use disorder and use of SNRI up to 7 days of index | X |  | X |
| depressive_7days_AND_family_therapy_7days | encounter with depressive disorder and family therapy up to 7 days of index | X | X | X |
| opiates_7days_AND_group_therapy_7days | encounter with opiate use disorder and group therapy up to 7 days of index | X | X | X |
| depressive_7days_AND_psych_ip_7days | encounter with depressive disorder and psychiatric hospitalization up to 7 days of index | X | X | X |
| thyroid_7days | encounter with thyroid disorder up to 7 days of index | X | X | X |
| SNRI_7days | use of SNRI up to 7 days of index | X |  | X |
| personal_safety_7days | encounter with expressed concern over personal safety up to 7 days of index | X | X | X |
| alcohol_7days_AND_medical_eval_observation_7days | encounter with alcohol use disorder and coded medical evaluation or observation up to 7 days of index | X | X | X |
| si_sa_7days_AND_gaba_a_agonist_7days | encounter with suicide ideation or attempt and use of GABA A agonist up to 7 days of index | X |  | X |
| psychotic_7days_AND_opi_antagonist_7days | encounter with psychotic illness and use of opiate antagonist up to 7 days of index | X |  | X |
| msld_7days | encounter with CCI-defined moderate or severe liver disease up to 7 days of index | X | X | X |
| opiates_7days_AND_medical_eval_observation_7days | encounter with opiate use disorder and coded medical evaluation or observation up to 7 days of index | X | X | X |
| nd_adhd_7days_AND_partial_opi_agonist_7days | encounter with ADHD and use of partial opiate agonist up to 7 days of index | X |  | X |
| ptsd_7days_AND_EM_99204_99215_4_7days | encounter with PTSD and EM code 99204 or 99215 up to 7 days of index | X | X | X |
| mh_counseling_7days | encounter with mental health counseling up to 7 days of index | X | X | X |
| ptsd_7days | encounter with PTSD up to 7 days of index | X | X | X |
| hp_7days | encounter with CCI-defined hemiplegia up to 7 days of index | X | X | X |
| alcohol_7days_AND_family_therapy_7days | encounter with alcohol use disorder and family therapy up to 7 days of index | X | X | X |
| depressive_7days_AND_gaba_a_agonist_7days | encounter with depressive disorder and use of GABA A agonist up to 7 days of index | X |  | X |
| opiates_7days_AND_tricyclic_antidep_7days | encounter with opiate use disorder and use of tricyclic antidepressants up to 7 days of index | X |  | X |
| si_sa_7days_AND_group_therapy_7days | encounter with suicide ideation or attempt and group therapy up to 7 days of index | X | X | X |
| fam_psych_7days | family history of psychiatric condition up to 7 days of index | X | X | X |
| alcohol_7days_AND_other_eval_px_7days | encounter with alcohol use disorder and other coded evaluation procedure up to 7 days of index | X | X | X |
| nd_adhd_7days_AND_OP_1mos | encounter with ADHD up to 7 days and outpatient services within 1 month of index | X | X | X |
| sedative_7days | encounter with sedative use disorder up to 7 days of index | X | X | X |
| si_sa_7days_AND_cns_stimulant_7days | encounter with suicide ideation or attempt and use of CNS stimulant up to 7 days of index | X |  | X |
| opiates_7days_AND_EM_99214_3_7days | encounter with opiate use disorder and EM code 99214 up to 7 days of index | X | X | X |
| anxiety_7days_AND_OP_1mos | encounter with anxiety disorder up to 7 days and outpatient services within 1 month of index | X | X | X |
| alcohol_7days_AND_gaba_a_agonist_7days | encounter with alcohol use disorder and use of GABA A agonist up to 7 days | X |  | X |
| metacanc_7days | encounter with CCI-defined metastatic cancer up to 7 days of index | X | X | X |
| bh_physical_activity_7days | encounter with coded observation suggesting diminished physical activity up to 7 days of index | X | X | X |
| anxiety_7days | encounter with anxiety disorder up to 7 days of index | X | X | X |
| count_ED_within_7days | count of ED encounters within 7 days of censored index date (e.g., 14 days total) | X | X | X |
| asthma_7days | encounter with asthma up to 7 days of index | X | X | X |
| anxiety_7days_AND_psych_ip_12mos | anxiety disorder up to 7 days and psychiatric hospitalization within 12 months of index | X | X | X |
| nd_adhd_7days_AND_EM_99204_99215_4_7days | encounter with ADHD and EM code 99204 or 99215 up to 7 days of index | X | X | X |
| legal_issues_7days | encounter with coded legal issues up to 7 days of observation | X | X | X |
| nd_adhd_7days_AND_cns_stimulant_7days | encounter with ADHD and use of CNS stimulant up to 7 days of index | X |  | X |
| nd_adhd_7days_AND_SSRI_7days | encounter with ADHD and use of SSRI up to 7 days of index | X |  | X |
| bipolar_7days_AND_tricyclic_antidep_7days | encounter with bipolar and use of tricyclic antidepressant up to 7 days of index | X |  | X |
| count_IP_within_7days | count of hospitalization encounters within 7 days of censored index date (e.g., 14 days total) | X | X | X |
| opiates_7days_AND_SSRI_7days | encounter with opiate use disorder and use of SSRI up to 7 days of index | X |  | X |
| alcohol_7days_AND_psych_ip_7days | encounter with alcohol use disorder and psychiatric hospitalization up to 7 days of index | X | X | X |
| financial_issues_7days | encounter with coded observation for financial issues up to 7 days of index | X | X | X |
| nd_adhd_7days_AND_psych_ip_7days | encounter with ADHD and psychiatric hospitalization up to 7 days of index | X | X | X |
| bipolar_7days_AND_group_therapy_7days | encounter with bipolar and group therapy up to 7 days of index | X | X | X |
| nd_adhd_7days_AND_gaba_a_agonist_7days | encounter with ADHD and use of GABA A agonist up to 7 days of index | X |  | X |
| opiates_7days_AND_psych_interview_exam_7days | encounter with opiate use disorder and psychiatric interview or exam up to 7 days of index | X | X | X |
| use_remission_7days | encounter with substance use disorder in remission up to 7 days of index | X | X | X |
| OP_12mos | outpatient service encounter within 12 months of index | X | X | X |
| alcohol_7days_AND_anti_epileptic_7days | encounter with alcohol use disorder and use of antiepileptic up to 7 days of index | X |  | X |
| nd_adhd_7days_AND_psychotherapy_or_addon_7days | encounter with ADHD and psychotherapy or psychotherapy add on up to 7 days of index | X | X | X |
| opiates_7days_AND_OP_7days | encounter with opiate use disorder and outpatient services up to 7 days of index | X | X | X |
| inhalant_7days | encounter with inhalant use disorder up to 7 days of index | X | X | X |
| bh_emotional_wellness_7days | encounter with coded observation suggesting poor emotional wellness up to 7 days of index | X | X | X |
| cerebrovascular_dis_7days | encounter with cerebrovascular disease up to 7 days of index | X | X | X |
| si_sa_7days_AND_SSRI_7days | encounter with suicide ideation or attempt and use of SSRI up to 7 days of index | X |  | X |
| hiv_aids_7days | encounter with HIV or AIDS up to 7 days of index | X | X | X |
| EM_99213_2_7days | encounter with EM code 99213 up to 7 days of index | X | X | X |
| chronic_pain_neuro_7days | encounter with chronic pain and neuropathy up to 7 days of index | X | X | X |
| anxiety_7days_AND_SNRI_7days | encounter with anxiety disorder and use of SNRI up to 7 days of index | X |  | X |
| bipolar_7days_AND_psych_ip_7days | encounter with bipolar and psychiatric hospitalization up to 7 days of index | X | X | X |
| bipolar_7days_AND_other_eval_px_7days | encounter with bipolar and other evaluation procedure up to 7 days of index | X | X | X |
| anxiety_7days_AND_cns_stimulant_7days | encounter with anxiety disorder and use of CNS stimulant up to 7 days of index | X |  | X |
| bh_nutrition_7days | encounter with coded observation suggesting poor nutrition up to 7 days of index | X | X | X |
| mld_7days | encounter with CCI-defined mild liver disease up to 7 days of index | X | X | X |
| decedent_age | age of decedent or patient as of index | X | X | X |
| IP_1mos | hospitalization within 1 month of index date | X | X | X |
| anxiety_7days_AND_EM_99204_99215_4_7days | encounter with anxiety disorder and EM codes 99204 or 99215 up to 7 days of index | X | X | X |
| si_sa_7days_AND_benzo_hypn_anxio_7days | encounter with suicide ideation or attempt and use of benzo/hypnotic or anxiolytic up to 7 days of index | X |  | X |
| psychotic_7days_AND_EM_99213_2_7days | encounter with psychotic illness and EM code 99213 up to 7 days of index | X | X | X |
| encs_within_7days | count of encounters within 7 days of censored index date (e.g., 14 days total) | X | X | X |
| si_sa_7days_AND_psych_interview_complex_7days | encounter with suicide ideation or attempt and complex psychiatric interview up to 7 days of index | X | X | X |
| myocardial_inf_7days | encounter with myocardial infarction up to 7 days of index | X | X | X |
| nd_adhd_7days_AND_benzo_hypn_anxio_7days | encounter with ADHD and use of benzo/hypnotic or anxiolytic up to 7 days of index | X |  | X |
| fam_substance_7days | family history of substance use disorders up to 7 days of index | X | X | X |
| lifestyle_manag_7days | encounter with coded observation suggesting lifestyle management issues up to 7 days of index | X | X | X |
| depressive_7days_AND_anticholinergic_7days | encounter with depressive disorder and use of anticholinergic up to 7 days of index | X |  | X |
| opi_antagonist_7days | use of opiate antagonist up to 7 days of index | X |  | X |
| heart_failure_7days | encounter with heart failure up to 7 days of index | X | X | X |
| psychotic_7days_AND_psychotherapy_or_addon_7days | encounter with psychotic illness and psychotherapy or psychotherapy addon up to 7 days of index | X | X | X |
| bipolar_7days_AND_psych_interview_exam_7days | encounter with bipolar and psychiatric interview or exam up to 7 days of index | X | X | X |
| peripheral_vasc_dis_7days | encounter with peripheral vascular disease up to 7 days of index | X | X | X |
| observation_rape_7days | encounter with observation following rape or assault up to 7 days of index | X | X | X |
| corticosteroid_7days | use of corticosteroid up to 7 days of index | X |  | X |
| mh_screening_7days | encounter with mental health screening up to 7 days of index | X | X | X |
| encs_within_12mos | count of encounter within 12 months of index | X | X | X |
| ptsd_7days_AND_satscan_5 | encounter with PTSD and resident of area cluster 5 up to 7 days of index | X | X |  |
| separated | marital status is separated at any time up to 7 days of index | X | X |  |
| nd_adhd_7days_AND_satscan_3 | encounter with ADHD and resident of area cluster 3 up to 7 days of index | X | X |  |
| medicaid_7days | Medicaid beneficiary at any time up to 7 days of index | X | X |  |
| nd_adhd_7days_AND_satscan_4 | encounter with ADHD and resident of area cluster 4 up to 7 days of index | X | X |  |
| Satscan_3 | resident of area cluster 3 up to 7 days of index | X | X |  |
| divorced | marital status is divorced at and time up to 7 days of index | X | X |  |
| psychotic_7days_AND_married | encounter with psychotic illness up and marital status is married at any time up to 7 days of index | X | X |  |
| bipolar_7days_AND_satscan_4 | encounter with bipolar and resident of area cluster 4 up to 7 days of index | X | X |  |
| si_sa_7days_AND_widowed | encounter with suicide ideation or attempt and marital status is widowed at any time up to 7 days of index | X | X |  |
| depressive_7days_AND_married | encounter with depressive disorder and marital status is married at any time up to 7 days of index | X | X |  |
| bipolar_7days_AND_satscan_5 | encounter with bipolar and resident of area cluster 5 up to 7 days of index | X | X |  |
| medicare_7days | Medicare beneficiary at any time up to 7 days of index | X | X |  |
| alcohol_7days_AND_separated | encounter with alcohol use disorder and marital status is separated at any time up to 7 days of index | X | X |  |
| opiates_7days_AND_separated | encounter with opiate use disorder and marital status is separated at any time up to 7 days of index | X | X |  |
| satscan_5 | resident of area cluster 5 up to 7 days of index | X | X |  |
| satscan_4 | resident of area cluster 4 up to 7 days of index | X | X |  |
| married | marital status is married at any time up to 7 days of index | X | X |  |
| widowed | marital status is widowed at any time up to 7 days of index | X | X |  |
| alcohol_7days_AND_satscan_2 | encounter with alcohol use disorder and resident of cluster 2 up to 7 days of index | X | X |  |
| psychotic_7days_AND_satscan_3 | encounter with psychotic illness and resident of cluster 3 up to 7 days of index | X | X |  |
| ptsd_7days_AND_widowed | encounter with PTSD and marital status is widowed at any time up to 7 days of index | X | X |  |
| anxiety_7days_AND_satscan_3 | encounter with anxiety and resident of cluster 3 up to 7 days of index | X | X |  |
| ptsd_7days AND_satscan_2 | encounter with PTSD and resident of cluster 2 up to 7 days of index | X | X |  |
| nd_adhd_7days_AND_separated | encounter with ADHD and marital status is separated at any time up to 7 days of index | X | X |  |
| private_7days | Private insurance beneficiary at any time up to 7 days of index | X | X |  |
| psychotic_7days_AND_separated | encounter with psychotic illness and marital status is separated at any time up to 7 days of index | X | X |  |
| anxiety_7days_AND_satscan_1 | encounter with anxiety disorder and resident of cluster 1 up to 7 days of index | X | X |  |
| anxiety_7days_AND_separated | encounter with anxiety disorder and marital status is separated at any time up to 7 days of index | X | X |  |
| alcohol_7days_AND_married | encounter with alcohol use disorder and marital status is married at any time up to 7 days of index | X | X |  |
| depressive_7days_AND_satscan_3 | encounter with depressive disorder and resident of cluster 3 up to 7 days of index | X | X |  |
| depressive_7days_AND_satscan_5 | encounter with depressive disorder and resident of cluster 5 up to 7 days of index | X | X |  |
| si_sa_7days_AND_married | encounter with suicide ideation or attempt and marital status is married at any time up to 7 days of index | X | X |  |
| opiates_7days_AND_married | encounter with opiate use disorder and marital status is married at any time up to 7 days of index | X | X |  |
| alcohol_7days_AND_widowed | encounter with alcohol use disorder and marital status is widowed at any time up to 7 days of index | X | X |  |
| alcohol_7days_AND_satscan_4 | encounter with alcohol use disorder and resident of cluster 4 up to 7 days of index | X | X |  |
| bipolar_7days_AND_satscan_3 | encounter with bipolar and resident of area cluster 3 up to 7 days of index | X | X |  |
| Satscan_2 | resident of area cluster 2 up to 7 days of index | X | X |  |
| ptsd_7days_AND_married | encounter with PTSD and marital status is married at any time up to 7 days of index | X | X |  |
| nd_adhd_7days_AND_satscan_5 | encounter with ADHD and resident of area cluster 5 up to 7 days of index | X | X |  |

**FIGURE CAPTIONS**

**Figure S1**. Selection of cohorts based on prospective time window
